# Single-Dilution COVID-19 Antibody Test with Qualitative and Quantitative Readouts

**DOI:** 10.1128/mSphere.00224-21

**Published:** 2021-04-21

**Authors:** Robert H. Bortz, Catalina Florez, Ethan Laudermilch, Ariel S. Wirchnianski, Gorka Lasso, Ryan J. Malonis, George I. Georgiev, Olivia Vergnolle, Natalia G. Herrera, Nicholas C. Morano, Sean T. Campbell, Erika P. Orner, Amanda Mengotto, M. Eugenia Dieterle, J. Maximilian Fels, Denise Haslwanter, Rohit K. Jangra, Alev Celikgil, Duncan Kimmel, James H. Lee, Margarette C. Mariano, Antonio Nakouzi, Jose Quiroz, Johanna Rivera, Wendy A. Szymczak, Karen Tong, Jason Barnhill, Mattias N. E. Forsell, Clas Ahlm, Daniel T. Stein, Liise-anne Pirofski, D. Yitzchak Goldstein, Scott J. Garforth, Steven C. Almo, Johanna P. Daily, Michael B. Prystowsky, James D. Faix, Amy S. Fox, Louis M. Weiss, Jonathan R. Lai, Kartik Chandran

**Affiliations:** a Department of Microbiology and Immunology, Albert Einstein College of Medicine, Bronx, New York, USA; b Department of Chemistry and Life Science, United States Military Academy at West Point, West Point, New York, USA; c Department of Biochemistry, Albert Einstein College of Medicine, Bronx, New York, USA; d Department of Pathology, Albert Einstein College of Medicine, Bronx, New York, USA; e Division of Infectious Diseases, Department of Medicine, Albert Einstein College of Medicine, Bronx, New York, USA; f Montefiore Medical Center, Bronx, New York, USA; g Department of Clinical Microbiology, Umeå University, Umeå, Sweden; h Division of Endocrinology and Diabetes, Department of Medicine, Albert Einstein College of Medicine, Bronx, New York, USA; University of Texas Southwestern Medical Center

**Keywords:** COVID-19, IgA, IgG, laboratory diagnostic test, quantitative test, SARS-CoV-2, serology, spike protein

## Abstract

The coronavirus disease 2019 (COVID-19) global pandemic caused by severe acute respiratory syndrome coronavirus 2 (SARS-CoV-2) continues to place an immense burden on societies and health care systems. A key component of COVID-19 control efforts is serological testing to determine the community prevalence of SARS-CoV-2 exposure and quantify individual immune responses to prior SARS-CoV-2 infection or vaccination. Here, we describe a laboratory-developed antibody test that uses readily available research-grade reagents to detect SARS-CoV-2 exposure in patient blood samples with high sensitivity and specificity. We further show that this sensitive test affords the estimation of viral spike-specific IgG titers from a single sample measurement, thereby providing a simple and scalable method to measure the strength of an individual’s immune response. The accuracy, adaptability, and cost-effectiveness of this test make it an excellent option for clinical deployment in the ongoing COVID-19 pandemic.

**IMPORTANCE** Serological surveillance has become an important public health tool during the COVID-19 pandemic. Detection of protective antibodies and seroconversion after SARS-CoV-2 infection or vaccination can help guide patient care plans and public health policies. Serology tests can detect antibodies against past infections; consequently, they can help overcome the shortcomings of molecular tests, which can detect only active infections. This is important, especially when considering that many COVID-19 patients are asymptomatic. In this study, we describe an enzyme-linked immunosorbent assay (ELISA)-based qualitative and quantitative serology test developed to measure IgG and IgA antibodies against the SARS-CoV-2 spike glycoprotein. The test can be deployed using commonly available laboratory reagents and equipment and displays high specificity and sensitivity. Furthermore, we demonstrate that IgG titers in patient samples can be estimated from a single measurement, enabling the assay’s use in high-throughput clinical environments.

## INTRODUCTION

The sudden emergence of severe acute respiratory syndrome coronavirus 2 (SARS-CoV-2), the causative agent of coronavirus disease 2019 (COVID-19), has resulted in ∼112.4 million cases and ∼2.5 million deaths worldwide to date (1, 2). SARS-CoV-2 is a member of the family *Coronaviridae*, which includes the endemic human coronaviruses (hCoVs) associated with mild respiratory illness and the highly virulent SARS and Middle East respiratory syndrome (MERS) coronaviruses (3, 4). Infection by SARS-CoV-2 is predominantly associated with mild to moderate flu-like symptoms (5, 6). However, like the SARS and MERS coronaviruses, SARS-CoV-2 can also cause severe respiratory disease (4–6). Current COVID-19 control efforts emphasize physical distancing, molecular testing for evidence of active infection, and isolation of infected and/or symptomatic individuals and their close contacts. Antibody testing to identify individuals with prior SARS-CoV-2 infection can complement these efforts. At the community and population levels, serological data can inform public health policy by uncovering spatial and temporal patterns of viral transmission. At the individual level, such testing is required to evaluate the kinetics and efficacy of the immune response to infection and vaccination. Thus, there is an urgent need for affordable and scalable antibody tests that provide both qualitative and quantitative data, ideally from single sample measurements, that can be widely implemented.

SARS-CoV-2 entry into host cells is mediated by the viral membrane-anchored spike glycoprotein (S), which forms homotrimers decorating the viral surface (7, 8). Endoproteolytic cleavage of the S precursor, largely by the proprotein convertase furin, liberates the S1 and S2 subunits and is necessary for virus-cell membrane fusion and cytoplasmic entry (7, 9–11). The S1 subunit mediates receptor binding and regulates the activity of the S2 membrane fusion subunit (7, 8). Mature viral spikes are a major target of the humoral immune response, and spike-specific antibodies that block viral entry into cells (neutralizing antibodies) can afford protection against severe disease (12, 13). A number of studies have shown that convalescent-phase patient sera contain high levels of SARS-CoV-2 spike-specific IgA, IgM, and IgG antibodies with significant neutralizing activity (14–17). In addition, the spike protein’s sequence divergence from those of the widely circulating endemic hCoVs (<30% sequence similarity of the S gene at the amino acid level) (18) makes it an ideal antigen to detect and measure SARS-CoV-2 seroconversion.

Here, we describe a highly sensitive and specific enzyme-linked immunosorbent assay (ELISA)-based test for SARS-CoV-2 exposure that was developed at the height of the COVID-19 pandemic in New York City in March to April 2020. The test employs a purified, recombinant SARS-CoV-2 spike protein ectodomain and readily available, research-grade laboratory reagents to detect spike-specific IgG and IgA antibodies in human sera. We show that the IgG test affords not only the qualitative assessment of SARS-CoV-2 exposure with high sensitivity and specificity but also the accurate determination of spike-specific IgG titers from a single sample measurement.

## RESULTS

### Development of an ELISA to detect SARS-CoV-2 spike-specific IgG and IgA in COVID-19 convalescent-phase sera.

Available serological assays for SARS-CoV-2 have used antigens derived from the spike and/or nucleocapsid proteins, which are the predominant targets of the humoral response to natural infection (7, 16, 17, 19, 20). Furthermore, many spike-specific assays have employed truncated forms of the spike protein (e.g., the S1 subunit or the receptor binding domain [RBD]) as the target antigen (16, 21, 22), in part because full-length spike can be challenging to produce at scale. Here, we utilized the full spike ectodomain as our assay antigen to test for antibodies that recognize all parts of the spike protein (19). Accordingly, we produced a previously described recombinant spike ectodomain protein bearing stabilizing mutations (8) using optimized expression and purification protocols that produce high yields of homogeneous, structurally well-defined spike trimers (23).

**FIG 1:**
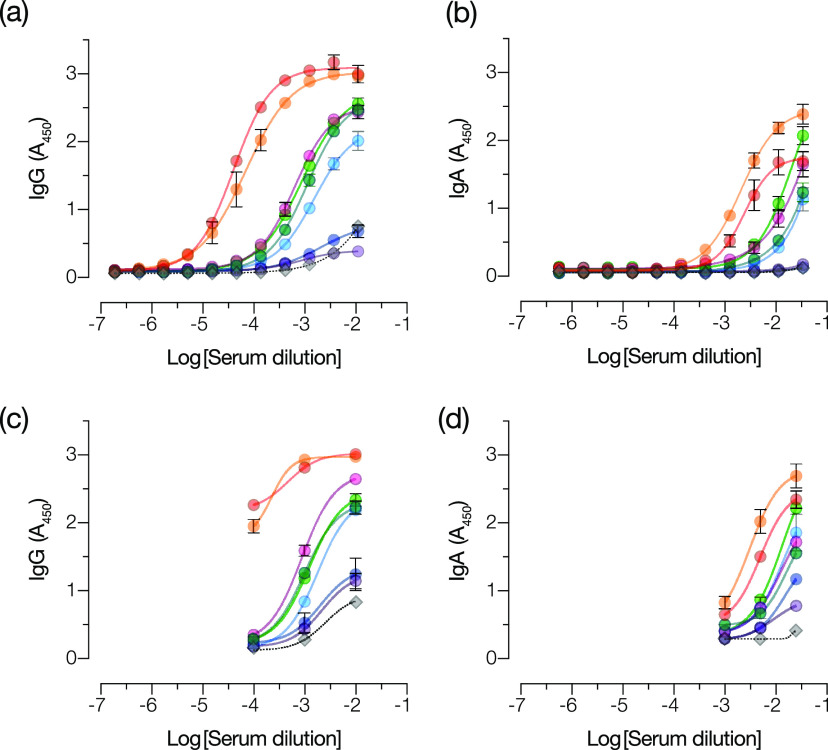
ELISA to detect and measure SARS-CoV-2 spike-specific IgG and IgA in COVID-19 convalescent-phase sera. Serially diluted convalescent-phase patient sera (colored circles) and a negative-control serum sample (gray diamonds and dotted lines) were added to recombinant SARS-CoV-2 spike protein-coated ELISA plates. (a and b) Captured IgG (a) and IgA (b) were detected using Ig class-specific secondary antibody-HRP conjugates. Absorbance (*A*_450_) values were fitted to a sigmoidal curve. (c and d) Samples were reanalyzed at three dilutions that best characterized the extent of the antibody reactivity for IgG (c) and IgA (d). Averages ± standard deviations (SD) are shown (*n* = 4 from two independent experiments). SD values smaller than the height of the symbols are not shown.

We examined the capacity of this trimeric spike protein to specifically capture antibodies in convalescent-phase sera from healthy individuals with prior SARS-CoV-2 infection. Spike protein-coated ELISA plates were incubated with serial dilutions of serum, and bound antibodies were detected and measured with a human IgG-specific secondary antibody conjugated to horseradish peroxidase (HRP). Various levels of spike-specific IgG were detected in convalescent-phase sera but not in a pre-COVID control serum sample (Fig. 1a).

Although most efforts to characterize the SARS-CoV-2 humoral immune response have focused on IgG, multiple reports suggest that IgA may be a sensitive marker for SARS-CoV-2 exposure and a marker for severe disease (14, 18, 24). Accordingly, we used the assay format described above but with a human IgA-specific secondary conjugate to detect and quantify spike-specific IgA in the same serum samples. IgA was consistently detected in these samples and was present at levels concordant with those of IgG (Fig. 1b).

### Definition of optimal single dilutions and corresponding diagnostic thresholds for the spike-specific IgG and IgA ELISAs.

**FIG 2:**
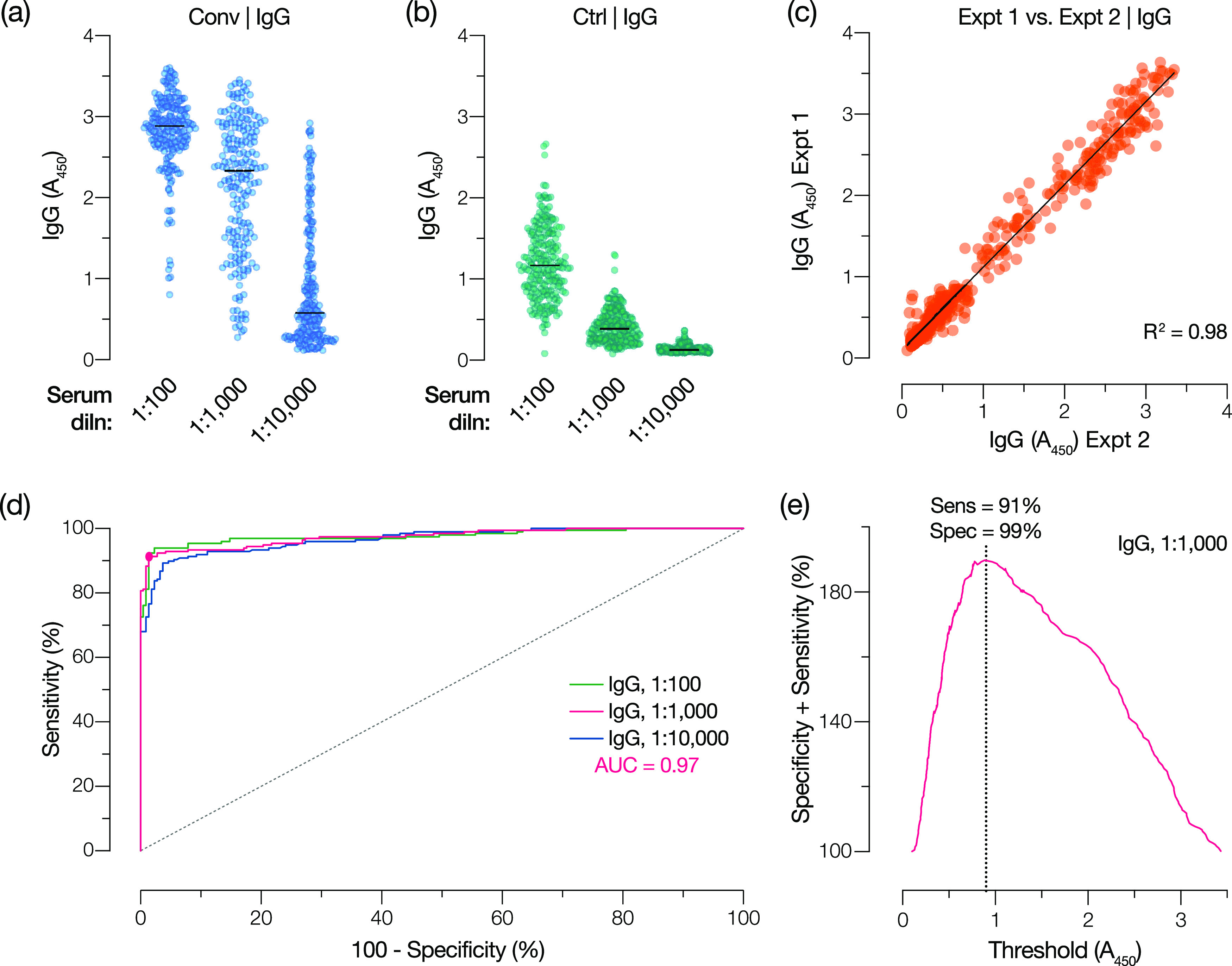
Spike-specific IgG reactivity in convalescent and control cohorts and receiver operating characteristic (ROC) analysis to select a single serum dilution and diagnostic threshold for the IgG test. (a and b) Spike-specific IgG responses at the indicated serum dilutions were determined for convalescent (Conv) (*n* = 197) and control (Ctrl) (*n* = 216) cohorts. (c) Interassay reproducibility of independent IgG assays at a serum dilution of 1:1,000 was assessed by linear regression analysis. (d) ROC analyses for the IgG test at serum dilutions of 1:100, 1:1,000, and 1:10,000 with the corresponding areas under the curve (AUC) for the 1:1,000 dilution. The filled circle indicates the point on the ROC curve that corresponds to the selected diagnostic threshold. (e) The sum of assay sensitivity and specificity for each candidate diagnostic threshold for a serum dilution of 1:1,000 was extracted from the ROC curve for the IgG test. The dotted line indicates the selected threshold (*A*_450_ of 0.90).

**FIG 3:**
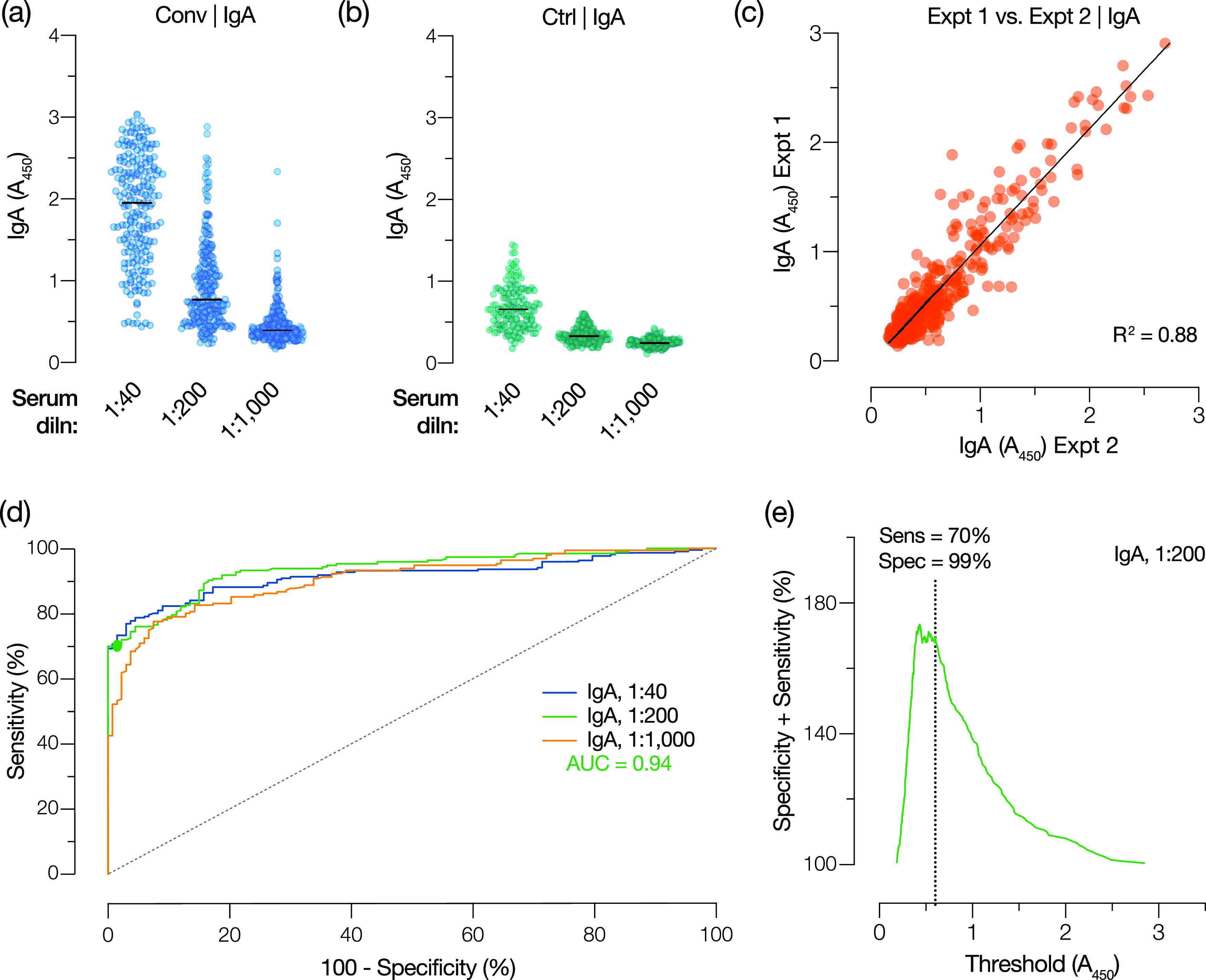
Spike-specific IgA reactivity in convalescent and control cohorts and ROC analysis to select a single serum dilution and diagnostic threshold for the IgA test. (a and b) Spike-specific IgA responses at the indicated serum dilutions were determined for convalescent (Conv) (*n* = 197) and control (Ctrl) (*n* = 216) cohorts. (c) Interassay reproducibility of independent IgA assays at a serum dilution of 1:200 was assessed by linear regression analysis. (d) ROC analyses for the IgA test at serum dilutions of 1:40, 1:200, and 1:1,000 with the corresponding AUCs for the 1:200 dilution. The filled circle indicates the point on the ROC curve that corresponds to the selected diagnostic threshold. (e) The sum of assay sensitivity and specificity for each candidate diagnostic threshold for a serum dilution of 1:200 was extracted from the ROC curve for the IgG test. The dotted line indicates the selected threshold (*A*_450_ of 0.60).

To develop the assay into a clinical laboratory test, we sought to identify a serum dilution that could provide a single threshold for reliably detecting spike-specific antibodies. Accordingly, we examined three sample dilutions each for IgG and IgA in an ELISA (Fig. 1c and d), which were selected from full response curves (Fig. 1a and b). Using this simplified three-dilution ELISA, we analyzed a large panel of sera from COVID-19 convalescent donors (Conv) (presumptively seropositive) and archival pre-COVID sera (control [Ctrl]) (presumptively seronegative) (Table 1) for both IgG (Fig. 2a and b) and IgA (Fig. 3a and b).

**TABLE 1:** Cohorts employed in this study^*l*^

Sample cohort	No. of individuals	No. of individuals of gender (M/F/NA)^*a*^	Age (yrs)^*b*^	No. of days after symptom onset^*b*^	No. of days after diagnosis^*b*^	Description
Conv	197	126/64/7	42 (32–54)	28 (24–31)	24 (20–27)	Mild disease, no O_2_ support

Hosp	27^*c*^	19/8	65 (55–73)	6 (1.5–7)^*c*^	0^*c*^	Moderate to severe disease
	27^*d*^			13 (10–16)^*d*^	8 (7–9)^*d*^	

Ctrl	45^*e*^	13/32^*e*^	54 (39–61)^*e*^	NA^*e*^	NA^*e*^	
	171^*f*^	53/103/15^*f*^	56 (49–62)^*f*^	NA^*f*^	NA^*f*^	

+Eval	50	34/16	63 (53–70)	17 (16–19)	11 (6–13)	Moderate to severe disease

−Eval	50	24/26	26 (16–36)	NA	NA	

Conv Follow Up	34^*g*^^,^^*h*^	23/11^*g*^^,^^*h*^	45 (38–58)^*g*^^,^^*h*^	31 (28–34)^*g*^	38 (30–42)^*g*^	Samples collected at 3 time points after symptom onset
	31^*i*^	21/10^*i*^	45 (38–59)^*i*^	98 (70–102)^*h*^	101 (73–106)^*h*^	
				178 (174–185)^*i*^	182 (178–193)^*i*^	

hCoV	17^*j*^	9/8^*j*^	66 (46–72)^*j*^	NA^*j*^	130 (31–221)^*j*^	Swab positive for OC43/HKU1, 229E, or NL63
	5^*k*^	3/2^*k*^	32 (24–37)^*k*^	NA^*k*^	NA^*k*^	

a M/F/NA, male/female/not available.

b Data are presented as medians (interquartile ranges).

c Samples collected 0 to 1 day after hospitalization.

d Samples collected 6 to 10 days after hospitalization.

e Samples collected from 28 to 30 January 2020.

f Samples collected from 2007 to 2019.

g Draw 1, samples collected ∼30 days after symptom onset, part of a larger convalescent-phase cohort.

h Draw 2, samples collected ∼100 days after symptom onset.

i Draw 3, samples collected ∼180 days after symptom onset. Not all patients returned for draw 3.

j Samples collected in Umeå, Sweden, in 2019 to 2020.

k Samples collected in Bronx, NY, in 2020.

l Conv, convalescent; Hosp, hospitalized; Ctrl, control; +Eval, positive evaluation; −Eval, negative evaluation; Conv Follow Up, convalescent-phase follow-up; hCoV, human coronavirus.

Individuals in the Conv cohort (*n* = 197) were initially selected to identify potential COVID-19 convalescent-phase plasma donors. Infection was confirmed by positive reverse transcription-quantitative PCR (RT-qPCR) for SARS-CoV-2 RNA during illness, and serum was collected after individuals had been asymptomatic for at least 14 days (median, 28 days after symptoms and 24 days after diagnosis). The Ctrl cohort was a set of patient serum samples collected at the Montefiore Medical Center (MMC) between 2008 and 2019 (Ctrl-Pre-2020) (*n* = 171) and in January 2020 (Ctrl-Jan 2020) (*n* = 45), prior to the identification of the first COVID-19 cases in the greater New York City area in late February 2020 (25) (Table 1). To assess assay reproducibility, the Ctrl and Conv samples were analyzed in two independent experiments conducted by different researchers. The average absorbance at 450 nm (*A*_450_) values from the independent experiments were found to be highly correlated for both IgG (Fig. 2c) and IgA (Fig. 3c).

The results from the seropositive and seronegative cohorts were analyzed using receiver operating characteristic (ROC) curves to determine assay sensitivity and specificity at each candidate threshold value (Fig. 2d, Fig. 3d, and Table 2). To maximize assay sensitivity and conserve clinical samples for additional laboratory tests, we selected the intermediate dilutions (1/1,000 for IgG and 1/200 for IgA) for further analysis (Table 2).

**TABLE 2:** Receiver operating characteristic analysis of SARS-CoV-2 spike-specific IgG and IgA antibody tests at three different dilutions of control and convalescent-phase antisera

Antibody class	Serum dilution	No. of positive samples^*a*^	No. of negative samples^*b*^	AUC^*c*^ (95% CI)^*d*^	Sensitivity (%) at 99% specificity^*e*^ (95% CI)^*d*^	*P* value
IgG	1:100	197	216	0.98 (0.96–0.99)	81 (75–86)	<0.0001
IgG	1:1,000	197	216	0.97 (0.96–0.99)	91 (87–95)	<0.0001
IgG	1:10,000	197	216	0.97 (0.95–0.98)	72 (65–78)	<0.0001
IgA	1:40	197	133	0.92 (0.89–0.95)	70 (64–76)	<0.0001
IgA	1:200	197	133	0.94 (0.91–0.96)	70 (63–76)	<0.0001
IgA	1:1,000	197	133	0.91 (0.88–0.94)	52 (45–59)	<0.0001

a Convalescent-phase donor cohort.

b Pre-COVID cohort (Ctrl).

c AUC, area under the curve.

d CI, confidence interval.

e Test sensitivity at 99% specificity.

**FIG 4:**
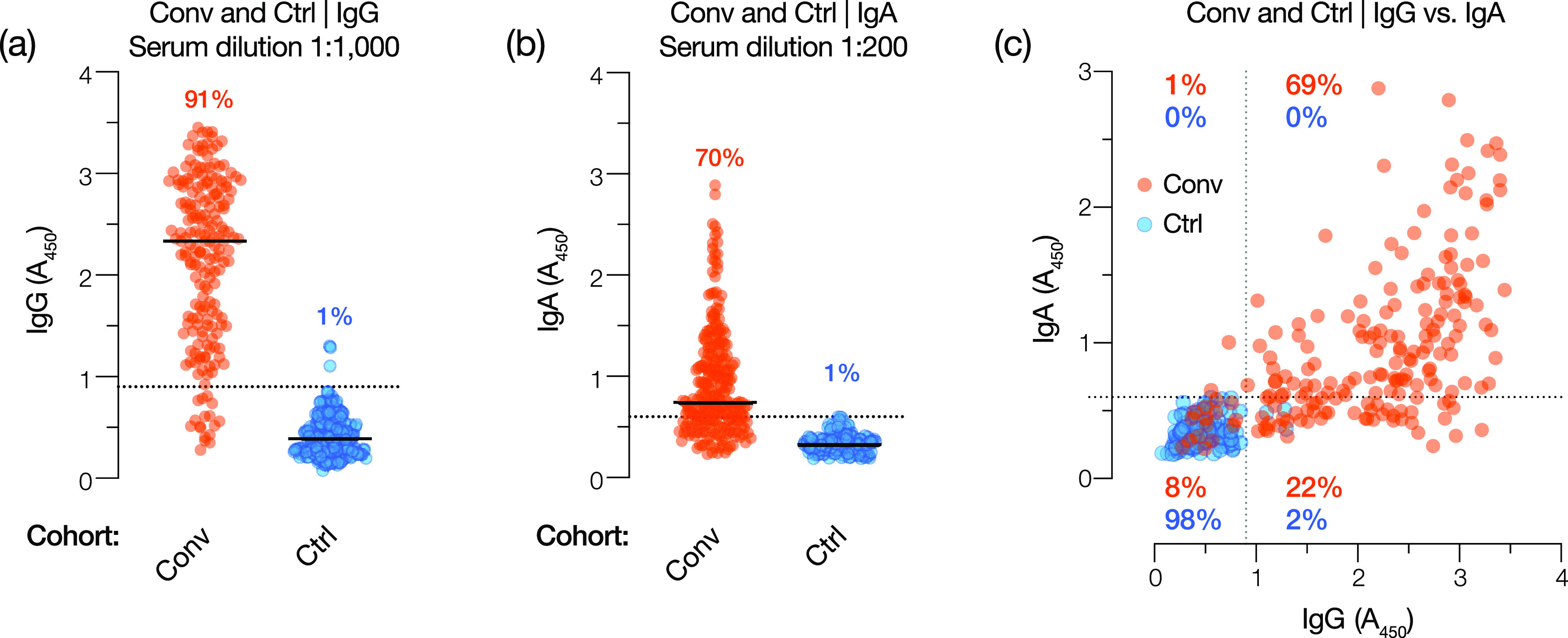
IgG and IgA test performance in Conv and Ctrl cohorts using the selected serum dilution and diagnostic threshold. (a and b) Spike-specific IgG and IgA reactivity for Conv (orange circles) and Ctrl (blue circles) cohorts at the selected test dilutions (1:1,000 and 1:200 serum dilutions, respectively). Diagnostic thresholds for IgG and IgA tests are shown as dotted lines (*A*_450_ values of 0.90 and 0.60, respectively). Percentages reflect the proportions of positive samples in each cohort (*A*_450_ above the threshold). (c) Comparison of the IgG and IgA reactivities of each sample in the Conv and Ctrl cohorts. The respective diagnostic thresholds are indicated as dotted lines. Percentages reflect the proportions of Ctrl and Conv samples in each quadrant.

To minimize the number of clinically harmful false-positive results, we selected a point on each ROC curve corresponding to a specificity of ∼99%, thereby obtaining threshold *A*_450_ values of 0.90 and 0.60 for IgG and IgA, respectively. These threshold values were at or near the maximal point of the curve comparing the sum of sensitivity and specificity against each candidate threshold, indicating nearly optimal assay performance (Fig. 2d and Fig. 3d). Reanalysis of the data sets at these thresholds yielded sensitivities of 91% and 70%, respectively, and a specificity of ∼99% for the IgG and IgA tests (Fig. 2e and Fig. 3e). By the application of these thresholds to the Conv and Ctrl groups, we find that 91% of the Conv cohort and 1% of the Ctrl cohort are positive for IgG and that 70% of the Conv cohort and 1% of the Ctrl cohort are positive for IgA (Fig. 4a and b and Tables 3 and 4).

**TABLE 3:** Results of the SARS-CoV-2 spike-specific IgG test

Sample cohort^*a*^	No. of positive samples	No. of negative samples	Total no. of samples	% positive samples	% negative samples
Conv	180	17	197	91	9
Ctrl-2020 (Jan 2020)	1	44	45	2	98
Ctrl-Pre (pre-2020)	2	169	171	1	99
hCoV	0	22	22	0	100
All controls	3	235	238	1	99

a Samples analyzed at a 1/1,000 serum dilution.

**TABLE 4:** Results of the SARS-CoV-2 spike-specific IgA test

Sample cohort^*a*^	No. of positive samples	No. of negative samples	Total no. of samples	% positive samples	% negative samples
Conv	138	59	197	70	30
Ctrl-2020 (Jan 2020)	0	45	45	0	100
Ctrl-Pre (pre-2020)	1	87	88	1	99
hCoV	0	22	22	0	100
All controls	1	154	155	1	99

a Samples analyzed at a 1/200 serum dilution.

Given the low sensitivity of the IgA test relative to the IgG test, we determined the relationship between the test results for each patient in the Conv and Ctrl cohorts (Fig. 4c and Table 5). Although IgG positivity correlated with that of IgA, especially for the strongly positive sera, a considerable proportion (22%) of the IgG-positive Conv sera were negative for IgA. The converse was not true: only 1% of Conv samples were positive for IgA but negative for IgG. These findings are consistent with emerging evidence that serum IgA wanes more rapidly than IgG in COVID-19 convalescent patients (26). We conclude that IgG provides a more sensitive probe of SARS-CoV-2 exposure in convalescent-phase patient sera than IgA when the full spike ectodomain is used as the capture antigen. Therefore, we focused our efforts on the further development of the anti-S IgG test.

**TABLE 5:** Results of SARS-CoV-2 spike-specific IgG→IgA tests

Sample cohort (no. of samples)^*a*^	No. of samples with result				% positive samples	% negative samples
	IgG^−^ IgA^−^	IgG^+^ IgA^−^	IgG^−^ IgA^+^	IgG^+^ IgA^+^		
Conv (197)	15	44	2	136	92	8
Ctrl (155)	152	3	0	0	2	98

a Samples analyzed at 1/1,000 and 1/200 serum dilutions for IgG and IgA, respectively.

### Assay performance in SARS-CoV-2-positive and -negative evaluation groups.

**FIG 5:**
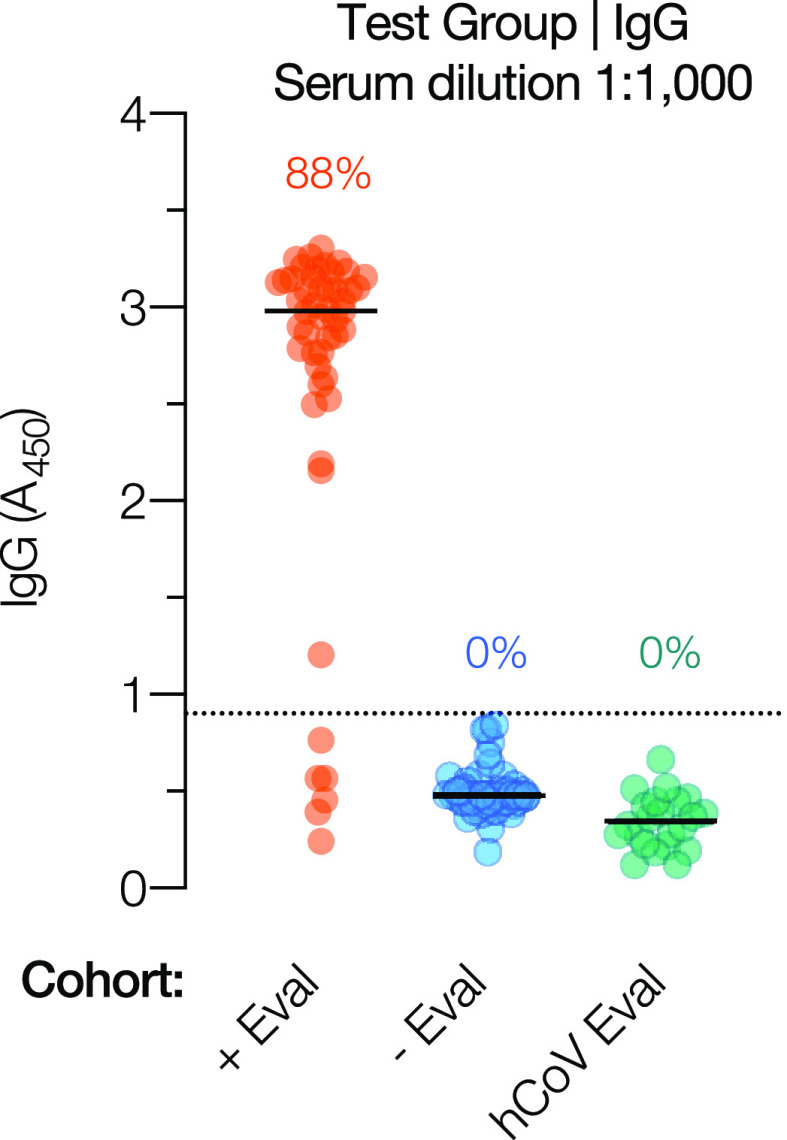
IgG test performance in an evaluation cohort. IgG reactivities in serum collected from hospitalized SARS-CoV-2-positive patients 14 to 21 days after symptom onset (+Eval) (*n* = 50) and serum collected from individuals prior to 2020 (−Eval) (*n* = 50) are shown. Data are from a single experiment (*n* = 2). Serum samples from COVID-19-negative patients with RT-qPCR-confirmed exposure to one or more commonly circulating human coronaviruses (hCoVs) were also analyzed for SARS-CoV-2 spike-specific IgG reactivity (hCoV Eval) (*n* = 22). Data from at least 2 independent experiments (*n* = 4 to 8) are shown for this cohort. Percentages reflect the proportions of positive samples in each cohort (*A*_450_ above the threshold indicated by the dotted line).

Following the establishment of a diagnostic threshold for the IgG test, we evaluated the test’s performance against positive evaluation (+Eval) and negative evaluation (−Eval) groups of serum samples. The +Eval group consisted of 50 serum samples from hospitalized patients with RT-PCR-confirmed SARS-CoV-2 exposure, collected 15 to 20 days after symptom onset. The −Eval group consisted of 50 additional serum samples from the Einstein Biorepository collected prior to 2020. The assay performance was similar to those for the initial positive (Conv) and negative (Ctrl) test cohorts, with sensitivity and specificity of 88% and 100%, respectively (Fig. 5).

### Prior exposure to endemic human coronaviruses is not associated with false-positive results.

The high seroprevalence of endemic human coronaviruses (hCoVs) (>90% of adults over 50 years old) (27, 28) and the low positivity rates of the archival Ctrl specimens in the spike IgG test strongly suggested that the test specifically detects the antibody response to the divergent SARS-CoV-2 spike protein. To further address SARS-CoV-2 specificity for the IgG assay, we tested 22 pre-COVID-19-pandemic serum samples (hCoV Eval) from individuals who had RT-qPCR-confirmed infection with hCoVs (Fig. 5). All of the samples were negative by our SARS-CoV-2 IgG test. Thus, the test is highly specific for SARS-CoV-2 and unlikely to engender false-positive results due to prior patient exposure to circulating hCoVs.

### Test performance in a cohort of hospitalized COVID-19 patients at early time points.

**FIG 6:**
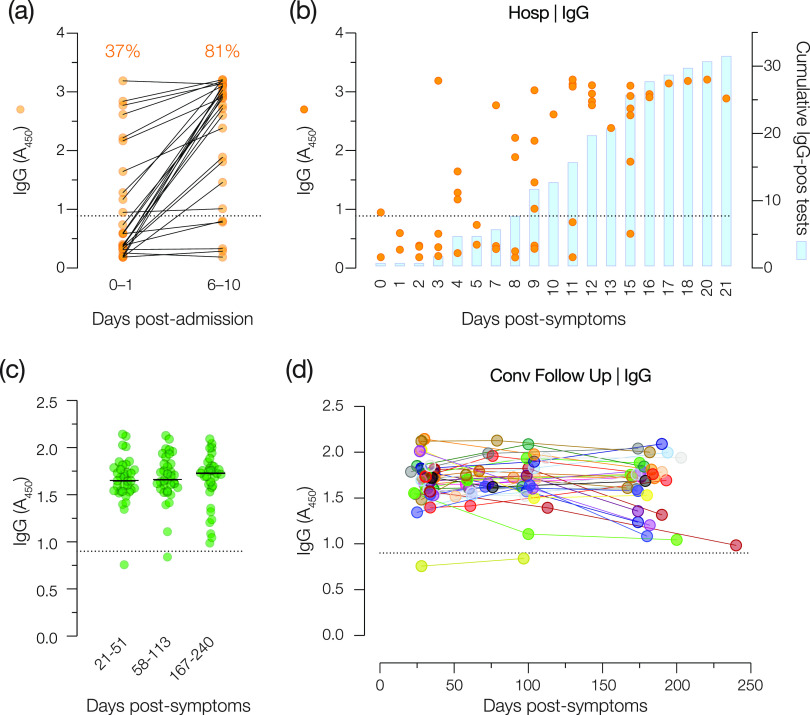
Longitudinal analysis of spike-specific IgG reactivity early in infection and up to 6 months after symptom onset. (a) Serum samples from patients at two time points following hospital admission were analyzed for spike-specific IgG. Percentages of positive samples are shown above each time point. (b) Individual patient samples (circles) and cumulative positive results (blue bars) graphed as a function of days after symptom onset. Data from two independent experiments (*n* = 4) are shown. (c and d) Serum samples collected from Conv cohort patients at two subsequent time points (∼100 and ∼180 days after symptom onset) were examined for spike-specific IgG reactivity. (c) Patient samples clustered by time of collection (days after symptom onset). (d) Longitudinal trends for individual patients. Data are from a single representative experiment (*n* = 2). The diagnostic threshold for IgG is depicted as a dotted line in each graph (*A*_450_ = 0.90).

We next assessed the capacity of the IgG test to detect SARS-CoV-2 exposure in recently hospitalized COVID-19 patients. The spike-specific IgG reactivities of blood drawn from each patient immediately (days 0 to 1) (early samples) after hospital admission or after 6 to 10 days (later samples) were determined. A total of 63% of the early samples were negative for IgG, whereas 81% of the later samples were positive (Fig. 6a), suggesting that most (but not all) of the patients developed a detectable antibody response to the SARS-CoV-2 spike protein over the first 6 to 10 days of their hospitalization. In relation to days after symptom onset, IgG was detected in at least some patients by day 8 and in a majority of the patients by day 14 (Fig. 6b).

### Test performance in convalescent patients at later time points.

Although some studies have observed a sharp reduction in anti-SARS-CoV-2 IgG titers early in the convalescent period (29, 30), others have observed a more durable response with only slight reductions in titers 6 to 8 months after symptom onset (31–33). To determine if decreases in antibody titers in the convalescent phase could cause patients with a prior positive IgG test result to drop below the diagnostic threshold of the test, we recruited convalescent patients from the Conv cohort to return and provide serum samples ∼60 and ∼120 days after initial symptom onset. All but 1 of these 34 individuals (who was negative at the initial sample collection) remained IgG positive (Fig. 6c and d).

### The IgG test affords quantitation of antispike antibodies from a single measurement.

**FIG 7:**
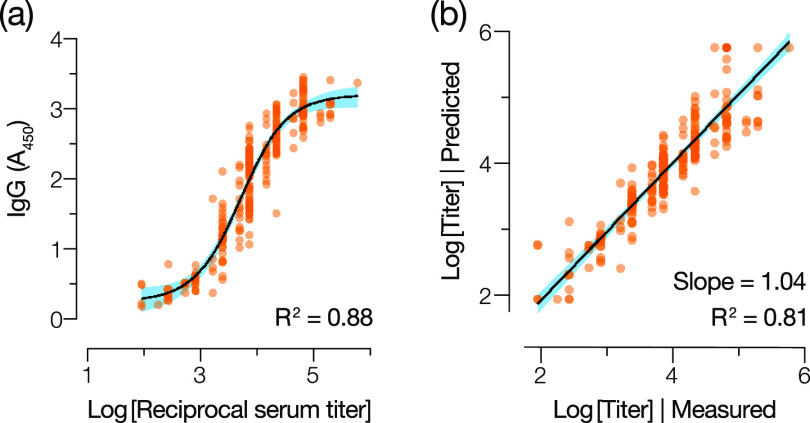
The IgG test affords quantitative assessment of serum IgG from a single measurement. (a) Relationship between the log-transformed readout value (*A*_450_ at a 1:1,000 serum dilution) in the IgG antibody test and the endpoint IgG titer (determined from full ELISA curves) for each serum sample in the Conv cohort. Data were fit to a sigmoidal function through nonlinear regression analysis. (b) A 10-fold cross-validation method was used to evaluate the predictive utility of this model. For each serum sample, the experimentally determined endpoint IgG titer was compared to that predicted from a single measurement with the antibody test using linear regression analysis. Shaded blue areas represent the 95% confidence intervals for the curve fits.

Having established and verified the assay performance of the IgG test, we investigated if it could also be utilized to determine a quantitative readout of the spike-specific antibody response in patient samples. We first generated serum titration curves (e.g., see Fig. 1) to determine spike IgG endpoint titers for the entire Conv cohort (*n* = 197). Next, we compared these titers to the independently measured *A*_450_ values for the same samples at a 1/1,000 dilution (Fig. 2a) and observed a nonlinear relationship (Fig. 7a). Accordingly, we modeled this relationship through nonlinear regression analysis by fitting a sigmoidal function using the least-squares method. Our model fit the experimental data well (Fig. 7a) (*R*^2^ = 0.88), suggesting that it could be used to infer spike-specific IgG titers from the single measurement performed for the diagnostic test. We further employed a 10-fold cross-validation method to evaluate the predictive utility of the model (see Materials and Methods for details). Our model could accurately predict the experimental IgG titer of a convalescent-phase serum sample based on a single *A*_450_ measurement (*R*^2^ = 0.81 ± 0.02) (Fig. 7b).

## DISCUSSION

As the COVID-19 pandemic continues, there remains a need for nonproprietary and scalable diagnostic antibody tests for monitoring populations that are vulnerable to SARS-CoV-2 and to gauge exposure at a population-wide level. High-throughput assays for quantitative serology are also urgently needed to support the development and global deployment of COVID-19 vaccines. Here, we describe and validate a simple, high-performance ELISA-based test for SARS-CoV-2 spike-specific IgG, developed at the height of the COVID-19 pandemic in New York City in March to April 2020. We also explore the utility of a highly specific IgA-based test for SARS-CoV-2 exposure. Finally, we demonstrate that our test can accurately quantitate SARS-CoV-2 spike-specific IgG in clinical samples from a single measurement.

The spike protein is a major target of the human antibody response to natural coronavirus infection and has key advantages as a capture antigen in serological assays. First, antispike antibody titers in convalescent-phase sera are related to antiviral neutralizing activity (16, 17, 34), decreased disease and viral loads in animal models (12, 13, 35–39), and survival following SARS-CoV-2 infection (12, 13). Second, the spike gene has the most divergent protein-coding sequence among the coronaviruses and, thus, is the least likely to engender false-positive results due to antibodies arising from endemic hCoV exposure (18, 40). Despite these potential advantages, the nucleocapsid protein has long been favored over spike in coronavirus serological assays, in part because it can be readily expressed at high levels without compromising conformation or immunogenicity (41). In contrast, prefusion trimers of the larger, more complex, and heavily glycosylated spike protein are more challenging to produce at scale (42). A number of spike-specific antibody tests have relied on individual spike subunits (typically, the highly immunogenic N-terminal subunit S1) (21, 43) or truncated protein fragments (typically, the receptor binding domain [RBD]) (16). We optimized the production and purification of a stabilized, full-length spike ectodomain described previously by Wrapp and colleagues (8) and showed that these scaled-up preparations largely consisted of homogeneous prefusion trimers (23). We leveraged these large, biochemically well-defined preparations to develop a scalable serological assay for SARS-CoV-2 that could comprehensively sample the antibody response to its spike protein.

We initially sought to establish a qualitative antibody test based on a standard ELISA format. We showed that analysis at three serum dilutions could corroborate the results of full antibody titration curves for IgG and IgA (Fig. 1). Further analysis of convalescent, pre-COVID control, and hCoV-exposed control cohorts at these three serum dilutions allowed us to identify optimal single dilutions and diagnostic thresholds for both tests. At the selected threshold, the IgG test was 91% sensitive and 99% specific for SARS-CoV-2, comparable to other highly sensitive spike-based assays (22, 44). The ROC analyses showed that further increases in sensitivity came at an unacceptable expense of specificity, validating the chosen threshold. Importantly, the test was comparably sensitive (88%) and specific (100%) for SARS-CoV-2 in a secondary evaluation cohort (Fig. 5). Although the failure of the IgG test to detect spike-specific antibodies above the threshold in ∼10% of COVID-19 convalescent patients (at an average of 28 days after symptom onset) may arise in part from technical limitations, it likely also reflects meaningful biological heterogeneity in the antibody response to natural infection (16, 45, 46). Our positive Conv cohort was composed solely of individuals characterized as having mild disease, with none requiring oxygen support. Recent work has shown that such individuals are more likely to seroconvert slowly and to have a lower overall antibody response (14, 18, 30, 47, 48).

The IgA test was considerably less sensitive (∼70%) than the IgG test at a threshold selected to provide 99% specificity (Fig. 3d and e). This differs from previous reports of antispike IgA assays that had higher sensitivity but lower specificity than IgG assays (14, 18, 21, 24, 49). This is unlikely to be due to the delayed development of an IgA response relative to IgG, as the kinetics of IgA seroconversion has been shown to resemble, or even slightly precede, that of IgG (14, 24). Rather, it may reflect the more rapid waning of serum IgA in convalescent patients (26). We also examined the possibility that, despite its lower sensitivity, the IgA test could be used to identify positive samples missed by the IgG test. We found that only 1% of the Conv cohort was positive for IgA alone, which was similar to the false-positive rate (Fig. 4c; Table 4). We conclude that there is no added value to combining the IgG and IgA tests or using the latter for reflex testing to diagnose SARS-CoV-2 exposure. Spike-specific IgA may nevertheless be of use as a biomarker to help assess disease severity in acutely infected patients (14, 24).

The performance characteristics of the IgG test were used to assess its clinical utility at different levels of population seroprevalence (Table 6). The seroprevalence in New York City was ∼20% at the end of April (50, 51). Furthermore, data from the New York City Department of Health and Mental Hygiene show that the seropositivity rate in Bronx County was 32.5% based on testing of over 17% of the county’s population (52). The seroprevalence at MMC, obtained during patient intake from 4 April through 27 August 2020, was 25.1% of 26,397 tests, using the Abbott SARS-CoV-2 IgG assay (obtained using the SlicerDicer function of MMC’s Epic Electronic Medical Record [E. Cadoff, personal communication]). Under these conditions, the IgG test described here has high positive and negative predictive values (PPV and NPV, respectively) (97%), which strongly supports clinical deployment of the test.

**TABLE 6:** Positive and negative predictive values of the IgG test at different levels of seroprevalence

Prevalence (%) (*n* = 100,000)	Sensitivity^*a*^ (%)	Specificity^*b*^ (%)	No. of positive tests/no. of COVID^+^ samples^*c*^	No. of positive tests/no. of COVID^−^ samples^*d*^	PPV^*e*^ (%)	NPV^*f*^ (%)
1	91	99	860/1,000	990/99,000	48	99.9
10	91	99	8,600/10,000	900/90,000	91	99
20	91	99	17,200/20,000	800/80,000	96	98
30	91	99	27,300/30,000	700/70,000	98	96

a Sensitivity at a cutoff of 0.9.

b Specificity at a cutoff of 0.9.

c Calculated number of positive tests in the group of true-positive samples.

d Calculated number of positive tests in the group of true-negative samples.

e PPV, positive predictive value (likelihood that a positive test predicts a true positive).

f NPV, negative predictive value (likelihood that a negative test predicts a true negative).

Seroconversion analysis after infection or vaccine administration is a rapid and economical way to gauge protective immunity (33). For this reason, we wanted to test the ability of our IgG assay to detect spike-specific antibodies at different time points. In a hospitalized COVID-19 cohort, we observed seroconversion in some patients 8 days after symptom onset and in nearly all patients 14 days after symptom onset. A convalescent COVID-19 cohort was surveyed longitudinally, and all patients had detectable spike-specific IgG antibodies at 6 months postinfection. Only 1 patient out of 34 was considered IgG negative, but this patient had a weak initial antibody response and never crossed the diagnostic threshold of the assay. Together, these analyses highlight the capacity of this IgG assay to detect SARS-CoV-2 exposure within a wide time frame and support its broad clinical and research utility.

Finally, we used independent experimental data sets from >200 convalescent-phase sera to generate a logistic regression model for the accurate estimation of IgG titers from single absorbance values obtained with the IgG test. Although the model described here is specific to the instrument used to read absorbance values in our laboratory, the model can be readily generated for a different instrument through analysis of a standardized set of serum samples for IgG titer, absorbance in the IgG single-dilution test, and the provided program (https://github.com/chandranlab/Ig_titer_sigmoid_fit). These findings expand the research and diagnostic utility of the Einstein/MMC IgG test without sacrificing its simplicity and throughput. Specifically, we believe that our test will help meet the need for quantitative serology engendered by the development and deployment of spike-based vaccines and convalescent-phase plasma transfusion therapy for COVID-19 (22). Indeed, given the significant percentage of COVID-19 convalescent patients with low or negative serological reactivity in this study, the rapid but accurate measurement of antibody levels in plasma will be crucial for vetting plasma collected from convalescent donors (53, 54). Furthermore, the rapid measurement of serum IgG and/or IgA in a point-of-care setting may find utility in clinical decision-making, including patient selection for the administration of medications such as steroids or convalescent-phase plasma (55, 56) to treat COVID-19.

## MATERIALS AND METHODS

### Patient cohorts. (i) Control cohorts.

Control (Ctrl) cohorts included patient serum samples collected prior to the identification of the first case of COVID-19 in the United States (Ctrl-2020, 45 deidentified remnant sera from unique patients collected in January 2020; Ctrl-Pre-2020, 171 deidentified remnant sera from unique patients, collected between 1 October and 1 January in 2007 to 2019 and stored in the Einstein Biorepository). Samples collected during this time frame were chosen to enrich for samples from patients with non-COVID-19 respiratory viral illnesses. Samples were collected for a variety of studies, but those from studies that enrolled HIV-infected patients were excluded.

### (ii) Human coronavirus cohort.

The human coronavirus (hCoV) cohort included remnant sera from patients with confirmed positive RT-qPCR tests for HCoV-229E, HCoV-OC43, HCoV-NL63, or HCoV-HKU1. Five sera were collected in January and early February 2020 and were identified from remnant sera in the Montefiore Medical Center (MMC) Pathology Laboratory. Another 17 sera were from samples collected from patients in Umeå, Sweden, in 2019 to 2020. All patient samples collected in 2020 were confirmed negative for SARS-CoV-2 by RT-qPCR.

### (iii) Hospitalized cohort.

The hospitalized (Hosp) cohort included deidentified remnant sera from 27 MMC inpatients who had COVID-19 with positive nasal swabs for SARS-CoV-2 by PCR. Serum samples collected on days 0 to 1 (early) and days 6 to 10 (late) after hospital admission were selected for analysis. Clinical data indicating how long symptoms were present before admission to the hospital were available for most patients and were used to analyze samples by days after symptom onset (e.g., if a patient had a history of 5 days of symptoms, the specimen was treated as day 5 in this series).

### (iv) Convalescent (Conv) cohort.

Deidentified samples from 197 healthy adult volunteers in Westchester County, NY, who had recovered from COVID-19 were collected as indicated below. All patients had confirmed SARS-CoV-2 infection with documented positive RT-qPCR results. All patients were at least 14 days after the resolution of symptoms (≥30 days postinfection) at the time of collection.

### (v) Convalescent follow-up (Conv Follow Up) cohort.

A subset of subjects in the convalescent cohort (see above) had additional serum samples collected between days 58 and 113 (*n* = 34) and from the same subjects collected between days 167 and 240 (*n* = 31) after symptoms.

### (vi) Positive evaluation cohort.

The positive evaluation (+Eval) cohort included deidentified serum samples from 50 hospitalized patients with RT-PCR-confirmed SARS-CoV-2 infection, collected 15 to 20 days after symptom onset.

### (vii) Negative evaluation cohort.

The negative evaluation (−Eval) cohort included 50 deidentified remnant sera from unique patients, collected between 1 October and 1 January in 2018 to 2019 and stored in the Einstein Biorepository. Samples collected during this time frame were chosen to provide samples from patients with non-COVID-19 respiratory viral illnesses. Sample collection excluded HIV-infected patients.

### Sample collection and handling.

Conv and Hosp cohort sera were obtained by venipuncture (BD Vacutainer, serum), centrifuged, aliquoted, and stored at −80°C. Prior to analysis for antispike IgG and IgA, samples were heat inactivated for 30 min at 56°C and stored at 4°C. Samples were handled under biosafety level 2 (BSL-2) containment in accordance with a protocol approved by the Einstein Institutional Biosafety Committee. Historical serum samples (Ctrl and hCoV cohorts) were previously stored at −80°C. Aliquots were thawed, heat inactivated as described above, and stored at 4°C prior to analysis.

### Protein production and purification.

A pCAGGS plasmid encoding a mammalian codon-optimized, stabilized SARS-CoV-2 spike protein with C-terminal Twin Strep and 8×His tags (gift from Jason McLellan [8]) was transiently transfected into ExpiCHO-STM cells (catalog number A29127; Gibco, Gaithersburg, MD) (0.8 μg DNA per ml of ExpiCHO-STM culture) according to the manufacturer’s instructions. Cells were incubated at 37°C for 1 day and then at 32°C in a shaking incubator (125 rpm with 8% CO_2_) and fed according to the manufacturer’s high-titer protocol. The supernatant was harvested on day 12 by centrifugation at 3,700 × *g* for 20 min, adjusted to pH 8, and dialyzed overnight at 4°C in Tris buffer (50 mM Tris HCl [pH 8.0], 250 mM NaCl). The supernatant was incubated with Ni-nitrilotriacetic acid (NTA) resin for 2 h at 4°C before resin was collected into a column and washed with Tris buffer plus 20 mM imidazole. Spike protein was eluted with Tris buffer plus 250 mM imidazole. The eluant was concentrated in an Amicon Ultra-15 100,000 nominal molecular weight limit centrifugal filter unit (catalog number UFC9010; Millipore Sigma, Burlington, MA) and buffer exchanged by dialysis into Tris buffer. Protein was aliquoted, flash-frozen in liquid nitrogen, and stored at −80°C. Protein quality was confirmed by analytical size exclusion chromatography using a Superose 6 Increase 10/300 GL column (Cytiva, Marlborough, MA) before and after flash-freezing.

### Spike-specific IgG and IgA ELISAs.

Half-area ELISA plates (catalog number 3690; Corning, Corning, NY) were incubated overnight at 4°C with 25 μl per well of 2 μg/ml of purified SARS-CoV-2 spike protein. Plates were washed three times with 120 μl per well 1× PBS-T (1× phosphate-buffered saline [PBS] [pH 7.4] plus 0.1% [vol/vol] Tween 20) using a microplate washer (BioTek, Winooski, VT) before being blocked for 1 h at 25°C with 150 μl per well of 1× PBS-T plus 3% (vol/vol) milk (catalog number 170-6404; Bio-Rad). Serum was serially diluted in 96-well non-tissue-culture-treated round-bottom plates (catalog number 22991; Celltreat, Pepperell, MA) using 1× PBS-T plus 1% (vol/vol) milk (1% milk–PBS-T) as the diluent. Blocked ELISA plates were washed three times with 120 μl per well of 1× PBS-T, and 25 μl of diluted serum was then added to wells in duplicate. Plates were incubated for 2 h at 25°C before being washed three times with 120 μl per well of 1× PBS-T. Plates were then incubated for 1 h at 25°C with 25 μl of the following secondary antibody (1:3,000 in 1% milk–PBS-T): goat anti-human IgG-horseradish peroxidase (HRP) (catalog number 31410; Invitrogen, Carlsbad, CA) or goat anti-human IgA-HRP (catalog number A0295; Millipore Sigma). Plates were washed as described above, prior to development with 25 μl per well of an ultra-TMB (3,3′,5,5′-tetramethylbenzidine) ELISA substrate solution at room temperature (catalog number 34029; Thermo Scientific). Plates were incubated in the dark for 5 min before quenching the reaction with 25 μl per well of 0.5 M sulfuric acid (catalog number 339741; Millipore Sigma). The absorbance at 450 nm (*A*_450_) was measured using a Cytation 5 plate reader (BioTek).

### Nonlinear regression analysis.

We used nonlinear least-squares analysis to fit a sigmoidal function to the experimental data (log_10_ IgG titer and *A*_450_ using a 1/1,000 dilution of serum) (Fig. 7):

$$y\;=\;y_{\min}\;+\;(y_{\max}-y_{\min})/\{1+10^{\left[(\log_{10}{\text{EC}}_{50}-x)\text{×Hill}\right]}\}$$

where *y* corresponds to the absorbance; *y*_min_ and *y*_max_ are the minimum and maximum absorbances, respectively; EC_50_ is the IgG titer that gives half-maximum absorbance, *y*_max_; Hill describes the slope of the curve, and *x* is the log_10_ IgG titer.

To predict IgG titers for a given *A*_450_ value (measured using 1/1,000-diluted serum) we first inferred *A*_450_ using the fitted sigmoidal model for 10,000 IgG titers evenly spaced between the experimentally observed minimum and maximum IgG titers in our data set. We then identified the closest inferred *A*_450_ to the queried *A*_450_ value and interpolated the corresponding IgG titer. We evaluated our nonlinear model by 10-fold cross-validation, where the original data set is randomly partitioned into 10 equally sized subsets, and 1 of the subsets serves as the testing set, while the remaining 9 subsets are used for training the nonlinear model. This process is repeated 10 times, using subsets for testing and training each time to ensure that all data points in our data set have been used once for testing. We iteratively (1,000 iterations) evaluated our nonlinear model by 10-fold cross-validation, computing the *R*^2^ value between the observed and predicted IgG titers at each iteration. Nonlinear regression was performed using the SciPy library (57). The source code is available at https://github.com/chandranlab/Ig_titer_sigmoid_fit.

### Institutional review board statement.

Approval for the development and validation of the COVID-19 antibody test protocol, including the collection and use of the various serum sample cohorts (Conv, Hosp, Ctrl, +Eval, and −Eval cohorts and U.S. samples in the hCoV cohort), was obtained by the Institutional Review Board (IRB) of the Albert Einstein College of Medicine (IRB number 2020-11421, approved on 8 April 2020 and amended on 13 and 17 April 2020). The protocol approval for the collection of longitudinal serum samples (Conv Follow Up cohort) was obtained by the IRB of the Albert Einstein College of Medicine (IRB number 2016-6137 on 19 March 2020). Serum samples collected at Umeå University Hospital (Swedish samples in the hCoV cohort) were obtained from the biobank repository of the Public Health Agency of Sweden as stipulated in the regulations for the use of such material in diagnostic development and quality assessment (http://www.epn.se/media/1205/the_ethical_review_actt.pdf).

### Informed consent statement.

Informed consent was obtained from all patients in the follow-up Conv cohort. All other samples were collected for prior studies or were remnant sera deidentified at the source, and informed consent was not required.
